# Concept analysis of impressionability among adolescents and young adults

**DOI:** 10.1002/nop2.170

**Published:** 2018-07-10

**Authors:** Seok Hyun Gwon, Suyong Jeong

**Affiliations:** ^1^ College of Nursing University of Wisconsin‐Milwaukee Milwaukee Wisconsin USA; ^2^ College of Nursing Research Institute of Nursing Science, Seoul National University Seoul Korea

**Keywords:** adolescent, commercial, concept analysis, impressionability, or not‐for‐profit sectors, youth

## Abstract

**Aim:**

To report a concept analysis of impressionability among adolescents and young adults.

**Background:**

Adolescence and young adulthood are critical periods to establish health behaviour. Environmental determinants are associated with youth health risk behaviours. These populations are impressionable to a range of social and physical environmental factors.

**Design:**

Concept analysis.

**Methods:**

We selected 17 studies to review from Psych INFO, ERIC, MEDLINE, and Google Scholar as well as the University of Virginia library. We did not apply date limits. We included search terms: “adolescent”; “youth”; “young adult”; “impressionability”; and “impressionable.”

**Results:**

The defining attributes of impressionability among youths were a state where: (a) one is able to be susceptible to external stimuli; (b) one is incapable of reasonable judgement; and (c) one has a changeability to accept or modify one's attitude and behaviour. We identified antecedents, consequences, and cases of impressionability.

## 1 INTRODUCTION

Researchers have reported that a range of social and environmental factors were associated with adolescent and young adult health risk behaviours such as smoking, drinking, and sexual behaviour. Adolescence and young adulthood are critical periods for tobacco control in that most smokers initiate smoking during adolescence and continue the behaviour during young adulthood (U.S. Department of Health & Human Services, 2012). Those populations would be exposed to harmful effects of tobacco consumption throughout the rest of their lives if smoking is not quitted. Recently, researchers documented that environmental determinants such as exposure to advertisements of and accessibility to tobacco products were related to smoking initiation among adolescents (Gwon, Yan, Huang, & Kulbok, 2018; Paynter & Edwards, 2009) and that tobacco marketing mainly targeted young adults (Ling & Glantz, 2002). In a similar vein, alcohol use was reported to be associated with various environmental factors such as the location and density of alcohol retailers among adolescents (Truong & Sturm, 2009; West et al., 2010) and young adults (Scribner et al., 2008). Adolescents' risky sexual behaviours (having intercourse with multiple partners, consuming alcohol or drugs while having intercourse, and anal intercourse) and attitudes were associated with exposure to sexually explicit websites (Braun‐Courville & Rojas, 2009).

A question arises. Why are adolescents and young adults impressionable to health risk behaviours? Given that Kinder and Sears (1985) used impressionable years indicating that adolescents and young adults are vulnerable to change, it is considered that youths are sensitive and easily reactive to external environments and these characteristics may influence the formation of attitudes and behaviours.

Impressionability is a concept that has been used by researchers to mean vulnerability and sensitivity to an environment and the concept is found in literature on environment and health risk behaviours among young people in various disciplines. Reviewing literature related to vulnerability, researchers have attempted a concept analysis of vulnerability in older adulthood (Brocklehurst & Laurenson, 2008) and in early childhood (Mattheus, 2010). Dorsen (2010) conceptualized the vulnerability among homeless adolescents with a perspective stemming from an evolutionary approach. Although vulnerability is applicable to populations of any age, impressionability is usually limited to adolescent and young adult populations. However, no researchers have yet attempted to analyse the concept of impressionability during adolescence and early adulthood while differentiating it from vulnerability.

## 2 BACKGROUND

Environment, one of four metaparadigm domains in nursing, is defined as “the entity that exists external to a person or to humanity, conceived either as a whole or as that which contains many distinct elements” (Kim, 2010). Nursing researchers have been interested in the needs and activities of people, which dynamically interact with the external world in multiple dimensions (Kim, 2010; Neuman, 1982). The domain of environment can include physical, social, symbolic, and holistic components (Kim, 2010).

Social learning theory demonstrates that individuals form attitudes and behaviours under the influence of other people nearby (Bandura & McClelland, 1977). Social effect is a strong factor related to youth health risk behaviours such as tobacco use (Gwon & Jeong, 2016; Gwon et al., 2018) and alcohol use (Nash, McQueen, & Bray, 2005). These days, young people are socially active in both offline and online settings (Huang, Unger, et al., 2014). According to Lenhart et al. (2018), 94% of adolescents access the Internet using a mobile device and 71% of those use more than one social media website. Content about tobacco products, such as electronic cigarettes (Huang, Kornfield, Szczypka, & Emery, 2014; Luo, Zheng, Zeng, & Leischow, 2014) and water pipes (Guidry, Jin, Haddad, Zhang, & Smith, 2016), are widespread on social media settings these days.

The ecological model of health behaviour posits that individual health behaviour is determined by multiple levels of social and environmental influence (McLeroy, Bibeau, Steckler, & Glanz, 1988). Built environment (Sallis et al., 2006), one of the environmental factors in the ecological perspective of health behaviour, may include marketing elements for drug sales. Gwon, DeGuzman, Kulbok, and Jeong (2017) considered tobacco marketing in retailers and geographic distribution of the retailers as a built environmental factor related to adolescent tobacco use.

### 2.1 Aims

To this end, we analysed the concept of impressionability using a method devised by Walker and Avant (Walker & Avant, 2005). The objective of this study is to identify attributes that are essential to the concept of impressionability among youths. By examining the attributes of impressionability, we will provide useful information for the development of theories, policies, and practices for control over harmful environments related to health risk behaviours and for capacity building for reasonable decision‐making among youths.

### 2.2 Data sources

**Figure 1:**
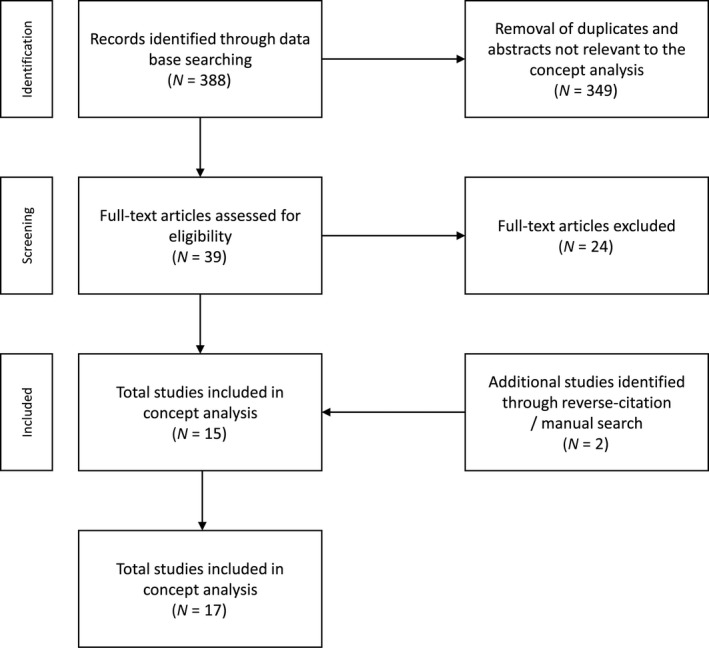
Flow diagram of the literature selection process

We searched empirical literature in the following databases: PsychINFO, ERIC, MEDLINE, Google Scholar, and the online catalogue of the University of Virginia library. Search terms included: “adolescent”; “youth”; “young adult”; “impressionable”; and “impressionability”. The search was conducted in 2017 and no date limits were applied. A total of 388 articles was initially searched for review. First, titles and abstracts of studies were reviewed for appropriateness for concept analysis. This limited the number of studies that appeared to match the aims of the concept analysis to 39. Full‐text articles were then assessed according to the inclusion and exclusion criteria. Inclusion criteria were studies: (a) including adolescents or young adults; (b) using the concept of being impressionable or impressionability; and (c) written in English. We excluded studies focusing only on children given the scope of this study. We manually included two studies from the University of Virginia library. Finally, 17 studies were included for the review (Figure 1).

### 2.3 Concept analysis method

Walker and Avant (2005) introduced the strategies for concept analysis. This method was derived from Wilson (1963) and initially consisted of 11 stages, but it was simplified to eight steps. The steps are: to (a) select a concept; (b) determine the aims or purposes of the analysis; (c) identify all uses of the concept; (d) determine the defining attributes; (e) identify a model case; (f) construct additional cases; (g) identify antecedents and consequences; and (h) define the empirical referents. We chose this method because our study aimed to identify attributes of impressionability among adolescents and young adults. Moreover, this method provides clear steps to follow and it has been evolved by several nursing researchers for evidence‐based nursing practice and research (Walker & Avant, 2005; Weaver & Mitcham, 2008). Because this study did not obtain private identifiable information and patient consent, Research Ethics Committee approval was not required.

## 3 RESULTS

### 3.1 Uses of concepts

The definitions of “impressionable” are present in dictionaries. Oxford University Press (2017) defines impressionable as “easily influenced,” and the American Heritage Dictionary (2017) defines it as “readily or easily influenced; suggestible or capable of receiving an impression.” Cambridge Dictionaries Online (2017) states that “impressionable” describes a person as being “easily influenced by other people, especially because you are young.” Synonyms for “impressionability” include: “sensitivity”; “impressibility”; “reactivity”; “receptiveness”; “sensitiveness”; “susceptibility”; “flexibility”; “perceptivity”; “plasticity”; and “pliancy” (Thesaurus.com, 2017). The word impressionable originated in the mid‐nineteenth century from French, from *impressionner*, which came from the Latin noun *impression*, derived from the verb *imprimere* (“press into”; Oxford University Press, 2017).

### 3.2 Theoretical uses of the concept

Unfortunately, impressionability has not been found in any theories. Although impressionability is not a term used very frequently in ordinary dialogue, it has been widely used in academia to describe the characteristics of youths. This concept was initially raised in the context of the “impressionable years” to explain a social change in psychology (Kinder & Sears, 1985; Krosnick & Alwin, 1989). Psychologists coined the concept of impressionability to describe the distinctiveness of late adolescence and early adulthood, which symbolize flexibility and openness to change.

### 3.3 Empirical uses of the concept

The concept of impressionability has been used in a range of disciplines (Table 1). Researchers have attempted to use impressionability to depict features of adolescents and young adults in a developmental context. Impressionability tended to be used primarily in psychology to investigate characteristics of these populations who are known to be interested in and easily influenced by current social issues.

**Table 1:** Empirical uses of impressionability in disciplines

Authors (Year)	Purpose	Concept attributes
Psychology		
Kinder and Sears (1985)	To provide an account of public opinion and political action congenial to social psychological tastes.	Flexibility and vulnerability in late adolescence and early adulthood in which people tend to change
Krosnick and Alwin (1989)	To test two hypotheses about the relation between age and susceptibility to attitude change.	Susceptibility to political attitude change during late adolescence and early adulthood
Tyler and Schuller (1991)	To examine the openness of people of varying ages to attitude change.	Openness to attitude change among younger people
Alwin et al. (1992)	To overview of political attitudes over the life span	Malleability of attitudinal orientations early in adult life
Silverstein et al. (2001)	To assess how the attitudes of Americans towards government programmes that serve older people changed and how much of the shift was due to intracohort change and how much was due to cohort replacement.	Susceptibility to public concern among young adults
May et al. (2004)	To examine the effects of an office workstation ergonomics intervention on employees' perceptions of workstation, and whether reactions differed between younger and older employees.	Being influenced among younger people
Education		
Carr et al. (2013)	To discuss developing first‐year students as scholars.	Being ready to learn. Optimal state for new experiences among college students
Medicine		
Glantz and Mandel (2005)	To discuss alternative methods for tobacco prevention among adolescents.	Susceptibility among youth
Miller (2005)	To report findings that neural plasticity can be extended into adulthood.	Malleability, adaptability, and plasticity to new experiences into adulthood
Beghi et al. (2006)	To introduce idiopathic generalized epilepsies of adolescence.	Being easily distracted in adolescence
Kligman et al. (2006)	To review experimental studies on the nature of sensitive skin.	Sensitivity to stimuli among female adults
Chuang et al. (2010)	To discuss the role of the “hidden curriculum” in shaping the professional identify of doctors in training.	Absorbency in learning among medical students
Nursing		
Hinkle and Kopp (2006)	To explore mentoring as a career development strategy for nursing students, minority nurses, and nursing faculty.	Imitation and internalization of others' characteristics among college students
Fenush and Hupcey (2008)	To investigate clinical unit choices of graduating baccalaureate nursing students and to identify factors that influence these decisions.	Being influenced by others among college students
Stretch et al. (2009)	To seek the views of school nurses on vaccinating girls who did not have parental consent.	Immaturity, being easily influenced, vulnerability, and being incapable of making a clear and independent judgement among 12–13 year olds
Walsh (2011)	To review of narrative pedagogy and simulation for nursing education.	Being distracted among college students
Fenwick et al., 2012	To examine that individuals will transform into three distinct types of decision‐makers using three different styles of decision‐making in response to the problems related to the experience of persistent pain.	Being easily influenced among individuals experiencing pain

Psychologists were inclined to use impressionability as a distinct and special characteristic of adolescent and early adult years. In 1985, impressionability was found in Kinder and Sears' (1985) study. They used “impressionable years” to refer to a flexible and vulnerable time to change attitudes among adolescents and young adults. It implies that impressionability has a feature of changeability. Krosnick and Alwin (1989) supported the concept of impressionable years of young people and they demonstrated that individuals in late adolescence and early adulthood are very susceptible to political attitude change even though their susceptibility drops rapidly thereafter. Similarly, Tyler and Schuller (1991) reported that impressionability is openness to attitude change among younger people in their study that examined attitude changes with various age groups. Alwin, Cohen, and Newcomb (1992) used impressionability to mean malleability of attitudinal orientations early in adult life. Silverstein, Angelelli, and Parrott (2001) used impressionability to mean susceptibility to public concern among young adults and May, Reed, Schwoerer, and Potter (2004) used the concept to mean the influence among younger people. Carr et al. (2013) used impressionability to mean the optimal state for new experiences among college students in an education article.

Moreover, impressionability has been used in the disciplines of medicine and nursing. Glantz and Mandel (2005) used it to mean susceptibility among youths. Miller (2005) used it to mean malleability, adaptability, and plasticity to new experiences into adulthood. Beghi, Beghi, Cornaggia, and Gobbi (2006) used it to mean easily distracted in adolescence, whereas Kligman Sadiq Zhen and Crosby (2006) used it to mean sensitivity to stimuli among female adults. Chuang et al. (2010) used it to mean absorbency in learning among medical students.

Hinkle and Kopp (2006) used impressionability to mean imitation and internalization of others' characteristics among college students. Fenush Jr. and Hupcey (2008) used impressionability to mean being influenced by others among college students. Stretch et al. (2009) used it to mean immaturity, being easily influenced, vulnerability, and being incapable of making a clear and independent judgement among 12–13 year olds. Walsh (2011) used it to mean being distracted among college students and Fenwick, Chaboyer, and St John (2012) used it to mean being easily influenced among individuals experiencing pain.

### 3.4 Defining attributes

Based on uses of impressionability, we identified a list of potential attributes of impressionability during adolescence and young adulthood:

1. Flexibility, susceptibility, openness, malleability, adaptability, plasticity, and vulnerability to attitude and behaviour change (Alwin et al., 1992; Beghi et al., 2006; Glantz & Mandel, 2005; Kinder & Sears, 1985; Krosnick & Alwin, 1989; Miller, 2005; Tyler & Schuller, 1991);
2. Susceptibility to public concerns (Silverstein et al., 2001);
3. Being easily influenced and distracted by an external environment (Beghi et al., 2006; Fenush Jr & Hupcey, 2008; Fenwick et al., 2012; May et al., 2004; Walsh, 2011);
4. Absorbency of learning (Carr et al., 2013; Chuang et al., 2010);
5. Imitating others (Hinkle & Kopp, 2006);
6. Sensitivity to stimuli (Kligman et al., 2006);
7. Immaturity and being incapable of making a clear and independent judgement (Stretch et al., 2009).

The identification of major attributes is the core step for concept analysis (Walker & Avant, 2005). After reviewing the literature and discussing potential attributes, the three defining attributes of impressionability were identified:

1. A state where one is able to be susceptible to external stimuli (from 2, 3, and 6 above);
2. A state where one is incapable of reasonable thinking (from 7 above);
3. A state where one has changeability to accept a new attitude/behaviour or change an existing attitude/behaviour (from 1, 4, and 5 above)

### 3.5 Model case

A model case is a paradigmatic exemplar that includes all defining attributes of the concept (Walker & Avant, 2005). The following model case was constructed by the authors. A 15‐year‐old boy named Tim lives with his parents and one brother. His father is a current cigarette user. Tim has a couple of close friends to spend spare time with. One friend, Ben, began to use electronic nicotine delivery systems (ENDS) two weeks ago. He described the positive feeling that ENDS use (vaping) led to. Tim was curious about the use of ENDS. One day, on the way home after a club activity, Tim saw Ben vaping. Ben said, “Do you want to give it a try?” Tim answered, “Why not?” Tim inhaled the ENDS for the first time in his life. He felt a warm vapour filling his airway and lungs. It tasted kind of sweet and fruity. The vaporization he exhaled was thick and beautiful. Whenever commuting to and from school, he would see a couple of vape shops that sell ENDS. The shops posted advertisements and messages that vaping is not harmful to one's health and not addictive at all. Tim believes that ENDS use is much less harmful than cigarettes; even if it is not, the harmful effects will not happen to him. He has a positive attitude towards vaping and wants to buy an ENDS some day from a vape shop.

All defining attributes of impressionability were found in this model case. Tim had susceptibility to external stimuli (his peer who vapes, his father who smokes, and messages from vape shops), incapability of reasonable judgement (wrong beliefs that harmful effects of ENDS will not happen to him), and changeability to accept or modify attitude or behaviour (positive attitude towards vaping).

Identifying additional cases such as borderline and contrary cases is helpful to clarify the characteristics of the concept (Walker & Avant, 2005).

### 3.6 Borderline case

An 18‐year‐old woman, Kate, is a new freshman in a state university. She does not like the smell of cigarette smoke because when she was a child her mother used cigarettes for more than 10 years. Kate is an outgoing and social person. She knows that recently vaping has had popularity among college students and she sees many students using ENDS on campus. She enjoys connecting to social media; whenever she gets on it, she frequently sees posts and photos related to ENDS. She hangs out with friends in shopping malls and she sees a lot of vape shops and vape stands that sell ENDS. She believes that ENDS use is not that bad for her health and that is the reason many people use ENDS. She thinks that ENDS use has nothing to do with addiction and it is easy to quit. She has a negative attitude towards ENDS use and has no plan to try it.

In this borderline case, only two attributes of impressionability can be seen: susceptibility to external stimuli (people who use ENDS and ENDS‐related content on social media) and incapability of reasonable judgement (misconception of harmful effects of ENDS).

### 3.7 Contrary case

A 17‐year‐old adolescent, Jake, is visually impaired. He lives with parents and one brother in a rural area of South Korea with few tobacco retail stores, vapes shops, and brick and mortar stores that sell tobacco products. He goes to a school for the blind. After school, he accesses the Internet to listen to video clips, music, and news and he reads books in braille and assists with house chores. His parents and friends are not smokers or ENDS users. Because advertising and posting any tobacco and ENDS‐related content on the Internet is not permitted, he is not exposed to tobacco or ENDS‐related promotions on the Internet. He took a health course from a licensed school nurse in school and learned that any tobacco including ENDS causes harmful effects on health. He has no interest in smoking and does not plan to try it.

This contrary case does not contain any attributes of impressionability. Rather, this is the complete opposite to impressionability.

### 3.8 Antecedents

**Figure 2:**
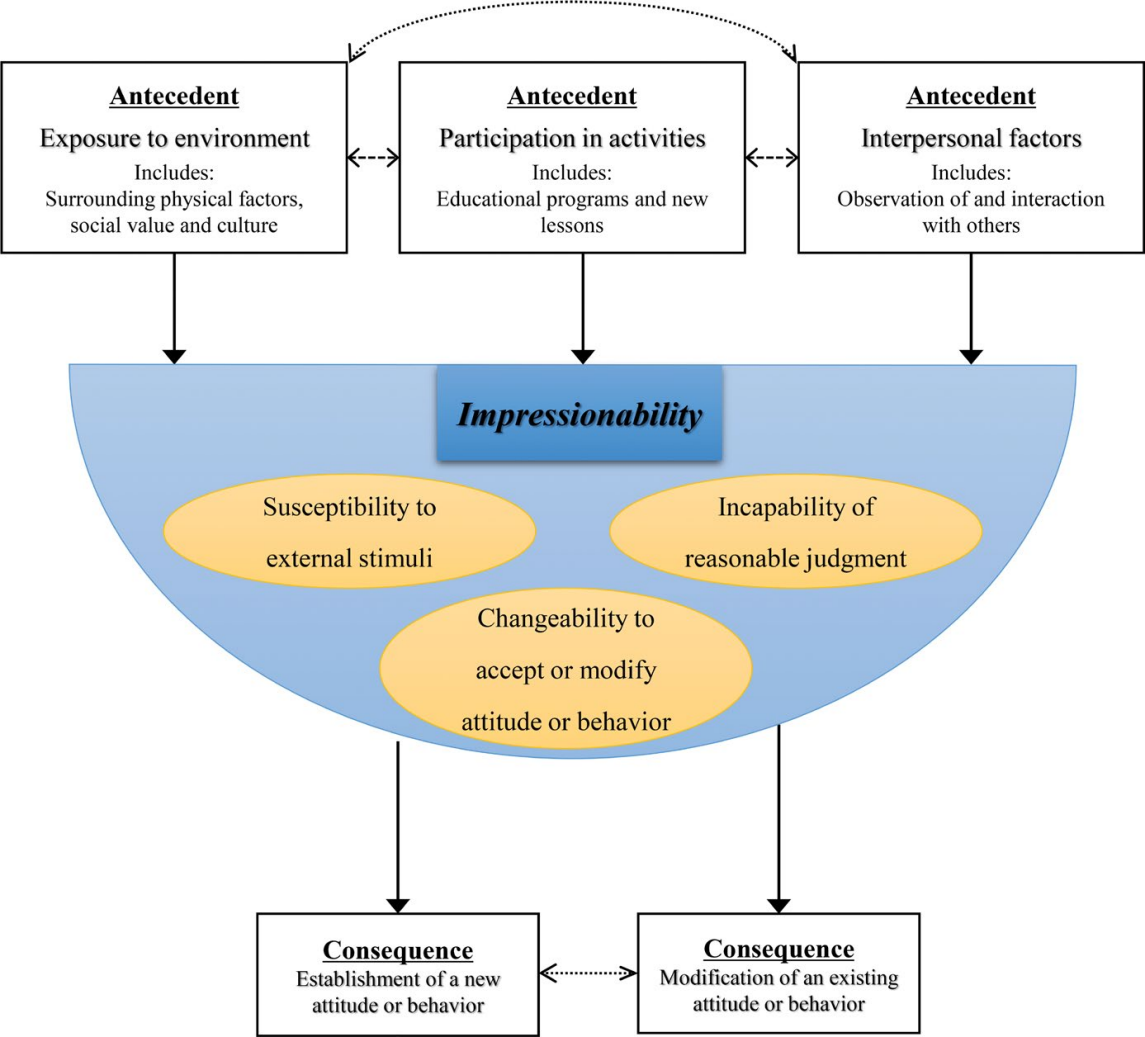
Conceptual map of impressionability among adolescents and young adults

Antecedents are the events that happen before a phenomenon occurs, whereas consequences are the results of a phenomenon (Walker & Avant, 2005). Prior to attainment of impressionability, one must be: (a) exposed to an environment–all of the certain circumstances that surround a person. This includes not only physical factors but also invisible factors such as social values or culture (Silverstein et al., 2001). The environment does not stand as it is, but rather exists with the potential to influence a person; (b) Participation in activities such as receiving an educational programme or new lesson is another antecedent to the appearance of impressionability (Carr et al., 2013; Glantz & Mandel, 2005); (c) Interpersonal factors arise prior to the induction of impressionability, including both observation of and interaction with others (Fenush Jr & Hupcey, 2008; Hinkle & Kopp, 2006) (Figure 2).

### 3.9 Consequences

Two consequences of impressionability were identified: (a) the establishment of a new attitude or behaviour; and (b) modification of an existing attitude or behaviour. Here, attitude refers to a disposition and perspective on an issue or behaviour. Behaviour refers to the actual actions of a person.

### 3.10 Empirical referents

According to Walker and Avant (2005), determining empirical references––the last stage of a concept analysis––is helpful for identifying the existence of the concept in real life. In other words, it proposes the standards by which to measure the concept. In many cases, the attributes and empirical referents of a concept are the same (Walker & Avant, 2005).

In this analysis, none of authors of the previous literature used empirical referents for the concept of impressionability. Reviewing the investigated attributes of impressionability, (a) susceptibility to external stimuli must be presented because the ability to sense an external environment is essential for impressionability. Researchers in studies on environmental determinants of youth health risk behaviour used items asking participants about the frequencies of exposure to harmful environmental factors such as advertisements, Internet posts, and retailers in regard to youth health risk behaviours (Gwon et al., 2018; Gwon, Yan, & Kulbok, 2017; Pokhrel et al., 2018). For (b), incapability of reasonable judgement, the examination of the knowledge, or perception of harmful effects of the health risk behaviours may be used. Specific age ranges such as from 12‐18 and 19‐24 years old may also be used to classify impressionable years. Researchers have used various instruments to measure (c), changeability to accept or modify attitudes and behaviour. Hanson (1997) measured attitudes towards smoking initiation and intention to smoke using the Fishbein/Ajzen‐Hanson Questionnaire. Participants rated attitude and intention on three 7‐point semantic differential evaluative scales of “pleasant/not pleasant”, “nice/awful”, “a lot of fun/not fun at all” and three other scales of “true/false”, “likely/unlikely”, “probably/probably not” with a range of scores from +3 ‐ −3 respectively. The sum of the first three scores for attitude was considered as the attitude towards smoking and the other three scores for intention were considered as smoking intention.

## 4 DISCUSSION

Concepts are essential parts of theories. A concept analysis is a paramount step prior to creating a nursing theory. If nursing theories effectively manage to describe phenomena of interest to the extent of predicting and controlling it, then nursing science will become a far more persuasive discipline. Adolescents and young adults are the primary target groups in population‐based community/public health nursing. Preventing health risk behaviours may play a key role for better health among youths because once a health behaviour is established, it is significantly hard to modify later in life.

The overarching goals of Healthy People 2020, the 10‐year plan outlining major national health objectives, include: (a) attaining high‐quality and longer lives free of preventable diseases, etc.; (b) accomplishing health equity and eliminating disparities; (c) forming healthy social and physical environments; and (d) promoting quality of life and healthy behaviours among all age groups (Office of Disease Prevention & Health Promotion, 2018). To this end, facilitating healthy behaviour and preventing health risk behaviours of the population are key components to improving health across the nation. Adolescence and young adulthood are critical periods in health behaviour intervention and policies. Many youths are exposed to harmful environmental factors such as online advertisements, video clips, and posts in regard to health risk behaviours and various physical environments including vape shops and liquor stores. There is a need to clearly understand and measure impressionability to evaluate the influence of harmful environments and susceptibility to the environments among youths. Disparities in health may exist among adolescents and emerging adults compared with general adults in that young populations are not mature enough to make right and optimal decisions for better health. These disparities may occur between homogeneous adolescent and young adult groups depending on defining attributes of the impressionability.

Health risk behaviours among adolescents and young adults have several special features such as being influenced by others (e.g., peers) and being easily susceptible to external environments (e.g., advertisements and content of tobacco products online as well as retailers) and inappropriate decision‐making caused by incomplete brain development (Dahl, 2004). Theories including social learning theory (Bandura & McClelland, 1977), ecological perspectives on health behaviours (McLeroy et al., 1988; Sallis et al., 2006), and empirical research study findings (Gwon et al., (2018); Gwon & Jeong, 2016) support this explanation.

Flaskerud and Winslow (1998) conceptualized vulnerability as the interaction between relative risk, resource availability, and health status in the vulnerable population model. Dorsen (2010) added in health perception to this model. According to this model, adolescents or young adults may be vulnerable populations because of high risks in health behaviour, lack of resources, and self‐perceived vulnerability. However, these models did not indicate susceptibility to environment and attitude and behaviour change.

Spiers (2000) demonstrated that the term *vulnerability* needs to be viewed from etic and emic perspectives. Spiers (2000) reported that attributes of the etic perspective of vulnerability include endangerment, functional capacity, external recognition, measurable behaviour, etc. and those of the emic perspective of vulnerability include integrity, perceived challenge, capacity for action, multidimensionality, power, and mutuality. Although these attributes of vulnerability are comprehensive, the number of attributes is high and it may be hard to conceptualize and measure all of the constructs.

Impressionability is different from vulnerability for these reasons. First, impressionable individuals must be exposed to external stimuli and they need to have susceptibility to the stimuli. For example, although the blind may be a vulnerable population because of a lack of resources and high risk of injuries and diseases, they may not be an impressionable population because of a lack of susceptibility to external stimuli. Second, impressionability should entail changeability to alter attitudes and/or behaviours. Vulnerability does not necessarily require attitude or action change. Impressionability results in the transformation of attitudes or behaviour by being influenced by and susceptible to various social and environmental factors. The attitude and behaviour established during adolescence and early adulthood hardly change late in adulthood.

We believe that the identification of attributes, antecedents, consequences, and models of impressionability in our study provided foundational information that will be helpful to create conceptual models and theories for adolescent and early adult health promotion and health behaviour modification. Policymakers, health providers, and health researchers involved in youth health may need to consider the findings of this study for the development and implementation of intervention programmes for healthy behaviours. If studies for the measurement of impressionability are followed, impressionability will be useful to prioritize and evaluate policies for the prevention of health risk behaviours and promotion of healthy environments among youths.

### 4.1 Limitations

This study has some limitations. First, this concept analysis used the method suggested by Walker and Avant (2005). Even though this method is widely used, it may be problematic in the use of concepts and differentiation of contexts (Yi et al., 2006). Second, this concept analysis focused only on adolescence and early adulthood. Further examination of impressionability for other developmental stages may provide more information that will be helpful to understand the entire context of impressionability. Third, we were not able to find empirical referents for impressionability. Possible reasons include an overlap in characteristics between vulnerability and impressionability, impressionability is only used for particular populations, impressionability is somewhat ambiguous for researchers to define, and measuring attributes of impressionability is not easy.

It is important to learn healthy behaviours and prevent health risk behaviours during adolescence and young adulthood. There is a need to examine additional perspectives on impressionability using other concept analysis methods such as the hybrid model and evolutionary method and focus on other developmental stages such as school ages. In addition, future studies need to develop an instrument or measures to evaluate impressionability for youths.

## 5 CONCLUSION

This study was the first attempt to examine the concept of impressionability during adolescence and early adulthood. The concept analysis was conducted in accordance with the method devised by Walker and Avant (2005). The literature was reviewed in disciplines of psychology, education, medicine, and nursing. A total of three critical attributes of impressionability were identified including susceptibility to external stimuli, incapability of reasonable judgement, and changeability to accept or modify attitude or behaviour. Antecedents were exposure to environment, participation in activities, and interpersonal factors. Consequences were the acquisition of new attitudes and behaviours and the modification of existing attitudes and behaviours.

## CONFLICT OF INTEREST

No conflict of interest has been declared by the authors.

## AUTHOR CONTRIBUTIONS

All authors have agreed on the final version and meet at least one of the following criteria (recommended by the International Committee of Medical Journal Editors [https://www.icmje.org/recommendations/]):

- substantial contributions to conception and design, acquisition of data, or analysis and interpretation of data;
- drafting the article or revising it critically for important intellectual content.
